# Friend or foe? Decoding the facilitative and disruptive effects of emotion on working memory in younger and older adults

**DOI:** 10.3389/fpsyg.2014.00094

**Published:** 2014-02-25

**Authors:** Linda Truong, Lixia Yang

**Affiliations:** Department of Psychology, Ryerson UniversityToronto, ON, Canada

**Keywords:** aging, emotions, working memory, goal relevance, distraction, interference resolution

## Abstract

A growing body of work on emotion-cognition interactions has revealed both facilitative and disruptive effects of emotion on working memory in younger adults. These differing effects may vary by the goal relevancy of emotion within a task. Additionally, it is possible that these emotional effects would be larger for older adults, considering findings of preserved emotional processing with age. To test these hypotheses, the current study examined the effects of emotional content and aging on working memory for target information in the presence of distraction. Thirty-six younger (ages 18–29) and 36 older adults (ages 65–87) completed a delayed-response working memory task. Participants viewed two target words intermixed with two distracter words, and then judged whether a subsequently presented probe word was one of the target words. The emotional content (valence and arousal) of targets and distracters was systematically manipulated. Results indicated that emotional targets facilitated working memory in both age groups. In contrast, emotional distracters disrupted performance. Negative distracters were particularly disruptive for older adults, but younger adults did not show an emotional interference effect. These findings help clarify discrepancies in the literature and contribute to the sparse research on emotional working memory in older adults.

## Introduction

The ability to successfully complete a task—such as driving—requires focused attention on task-relevant information, like traffic lights, and limited attention to task-irrelevant distraction, such as roadside advertisements. Similarly, successful working memory performance consists of briefly maintaining relevant target information while ignoring interference from irrelevant distraction. Research suggests that working memory declines with normal aging, resulting in slower and less accurate responses in older adults (e.g., Rypma and D'Esposito, 2000; Gazzaley et al., 2005). According to the inhibitory deficit theory, age-related deficits in inhibition—the ability to avoid or remove distracting information from working memory—reduces the ability to focus attention on task-relevant information, thus lowering overall working memory performance (i.e., slower and/or less accurate responses) (Hasher and Zacks, 1988). In contrast to this decline, processing of emotional information is preserved in old age (see Scheibe and Carstensen, 2010 for a review). A body of research suggests a tendency for older adults to attend to, and remember, positive information relative to neutral and negative information, termed an age-related “positivity effect” (Mather and Carstensen, 2005; see Reed and Carstensen, 2012 for a review). Several theories have been proposed to account for this age-related preference toward positive information (Murphy and Isaacowitz, 2008 for a meta-analysis that did not find evidence of this preference). According to the socioemotional selectivity theory (Carstensen et al., 1999), the preserved emotional processing and functioning in old age is due to a motivational shift toward emotional regulation goals (i.e., achieving positive affect) as a result of increasingly limited time horizons with age. The dynamic integration theory (Labouvie-Vief, 2003) proposes that older adults increasingly prefer positive over negative information, due to greater cognitive demands required to process the latter. Taken together, these findings raise an important question: how is working memory influenced by emotional information in older adults? Specifically, would older adults' preserved emotional processing lead to working memory enhancements or would it further impair performance?

Some insights into these questions can be gained from a theory about working memory and emotion interactions: the dual-competition model (DCM; Pessoa, 2008, 2009). This model states that biases toward emotional information influence the allocation of processing resources. When emotional information is relevant to a task goal, performance is facilitated due to the additional resources that are recruited for emotional processing. However, this bias toward emotional information can be impairing if it conflicts with a task goal, thus depleting resources needed for executive control processes in working memory, as in the case of task-irrelevant emotional information (e.g., emotional distracter items). Thus, DCM predicts that working memory performance will be disrupted in the face of task-irrelevant emotional information. The DCM can be used to interpret the results of studies that have examined emotional working memory in both older and younger adults. For example, one study compared maintenance of affective (emotional intensity of emotional images) vs. visual information (brightness intensity of neutral images) over a delay period with both younger and older adults (Mikels et al., 2005). Results revealed an age-related working memory deficit for visual maintenance, but no such deficit was found for maintenance of emotional intensity. Interestingly, older adults actually outperformed younger adults on trials where positive affect was maintained. In contrast, younger adults performed better on negative relative to positive trials. These results suggest that older adults' preserved emotional processing—of positive information, in particular—can offset their working memory declines, resulting in maintained or even enhanced performance. Similarly, another study by Mammarella et al. (2013a) found that age-related deficits in working memory were reduced when the task contained emotional information. This study examined performance on an operation span task in which participants maintained a set of neutral or emotional target words in working memory while performing math operations. Results indicated larger age differences for neutral words, but reduced or eliminated age differences for positive and negative words, respectively. A similar study also found better working memory performance for positive and negative words, relative to neutral words, in older adults (Mammarella et al., 2013b). Such findings also extend to paradigms using emotional pictures. Borg et al. (2011) compared performance on two working memory tasks with emotional pictures in younger and older adults. In the first task, participants were shown two negative and two neutral pictures, presented sequentially. The task was to maintain these four target pictures during a delay, after which a probe picture appeared. Participants had to indicate whether this probe was an old (i.e., from the target set) or a new picture they had not seen before. For both age groups, accuracy on this task was better when target stimuli were negative vs. neutral. Taken together, these findings showed little age differences in emotional working memory, suggesting that performance on task-relevant emotional information is relatively preserved in older adults.

It should be noted that previous studies have primarily focused on analyzing the effect of emotional task-*relevant* information—little attention has been paid to the potential effects of task-*irrelevant* emotional information. The few existing studies have been primarily conducted with younger adults. In one such study, it was found that emotional distracters presented during a delay period can impair younger adults' working memory performance for target items (e.g., Dolcos and McCarthy, 2006). It is unknown whether detrimental effects of emotion would be found or perhaps magnified for older adults. The second working memory task in Borg et al. (2011) sheds a light on this. In this task, participants were asked to bind four target pictures (negative or neutral) with their respective presentation locations and then identify whether a subsequently presented probe picture was presented in its original location. Results revealed no emotional effects on younger adults' performance. However, older adults performed worse for negative vs. neutral pictures. It was interpreted that resources devoted to processing negative information may have limited the availability of resources needed for effortful processing in working memory (i.e., binding) in older adults. Thus, the emotional aspects of the negative pictures may have diverted resources away from the primary binding task. However, another study did not find any effects of emotional pictures on a 2-back task (which consists of both target and distracter items) in older adults (Döhnel et al., 2008). Thus, it remains unclear when emotional information hinders older adults' working memory.

The current study took a novel approach to directly examine whether the effects of emotional content vary depending on the goal-relevancy of the emotional content. Examining the effect of emotional distracters is particularly important considering findings that older adults' working memory deficits are due to a specific decline in the inhibition of distracters, rather than the ability to attend to and maintain goal-relevant content (Gazzaley et al., 2005). In the current study, we modified the delayed-response working memory paradigm used by Gazzaley et al. (2005) in which both targets and distracters were presented within a memorandum set. Younger and older participants viewed four sequentially presented words: two were cued as targets and two were cued as distracters, by different colored fonts. After a delay, a probe word (could be a target, a distracter, or a new control probe) appeared. Participants' task was to indicate whether this probe was a word that was cued as a target from the current memorandum set. For some trials, targets were emotional words presented with neutral distracter words; for others, targets were neutral and distracters were emotional. These trials were compared to trials where both targets and distracters were neutral words, to evaluate the emotional effect of task-relevant (i.e., targets) vs. task-irrelevant (i.e., distracter) information. The critical manipulation was the emotional content—as indexed by both the valence (positive, negative, neutral) and arousal (high vs. low)—of targets and distracters. Arousal was manipulated in this study given the evidence of distinct neural networks involved in processing arousing vs. valenced information (Kensinger and Corkin, 2004) and differential effects of low vs. high arousal valenced information (Leclerc and Kensinger, 2008). The arousal levels of stimuli were systematically manipulated so that half of the words were high arousal and half were low arousal within each of the three valence categories. This allowed us to evaluate valence effects by controlling for the level of arousal.

This study aimed to address three questions: (1) Does emotional target information facilitate working memory? (2) Does emotional distracter information hinder working memory? (3) Do these effects change with age? Based on predictions derived from the dual-competition model (Pessoa, 2008, 2009) and the literature reviewed earlier, several hypotheses were proposed. First, it was expected that emotional content would be facilitative to working memory (i.e., faster and/or more accurate responses) for goal-relevant target information and would reduce age differences in working memory, in line with previous studies (e.g., Mikels et al., 2005; Mammarella et al., 2013a). In contrast, performance would be disrupted (i.e., slower and/or less accurate responses) by emotional distracters (e.g., Dolcos and McCarthy, 2006); we hypothesized that a detrimental effect due to emotional content would be more evident in older adults. Older adults' limited ability to inhibit distracter information (e.g., Yang and Hasher, 2007) may result in increased demand for cognitive resources to successfully resolve interference arising from these distracters. However, based on the dual-competition model, resources for interference resolution may be further limited when they are prioritized to be diverted toward the processing of emotional information (Pessoa, 2008, 2009). As such, we hypothesized that disruptive effects of emotional distracters would be more evident in older vs. younger adults. Finally, we also hypothesized that older adults would show enhanced attention to positive information, in line with findings of an age-related positivity bias (Mather and Carstensen, 2005). Specifically, we expected that this enhanced attention would result in facilitative effects of positive target information but also disruptive effects from positive distracter information.

## Materials and methods

### Participants

Thirty-six healthy younger adults (ages 18–29, *M* = 19.69, *SD* = 2.84; 3 males) and 36 healthy older adults (ages 65–87, *M* = 73.25, *SD* = 6.37; 6 males) participated in this study. Younger adults were recruited from the undergraduate participant pool at Ryerson University. They received course credit as compensation. Older adults were recruited from the Ryerson Senior Participant Pool at Ryerson University and received $10 per hour for participation. Four older adults were replaced: three for low accuracy in the working memory task (< 80%; see Results) and one due to computer malfunctions. All participants were tested at the Cognitive Aging Laboratory of Ryerson University and provided informed consent prior to commencing the study. All procedures in the study were conducted according to regulatory standards and were approved by the Research Ethics Board at Ryerson University.

We excluded participants who: (a) learned English after the age of 6; (b) scored less than 20 on the Shipley Institute of Living Vocabulary (Shipley, 1940); (c) scored greater than 26 on the Beck Anxiety Inventory (BAI; Beck et al., 1988), suggesting severe anxiety symptoms; (d) scored greater than 29 on the Beck Depression Inventory (BDI; Beck et al., 1996), suggesting severe depressive symptoms; (e) had previous neurological disorders (e.g., stroke, dementia, prolonged periods of unconsciousness, and head injury); or (f) uncontrolled medical conditions (e.g., diabetes, cholesterol, and cardiovascular diseases). Older adults were screened with the Short Blessed Test (SBT; Katzman et al., 1983) for dementia-related cognitive impairments and all participants in the final sample scored above the cut-off score of 6 (*M* = 0.78, *SD* = 1.35). All demographic and health information were collected through a background information questionnaire. There were age differences in several demographic and cognitive measures (see Table 1). Older adults had more years of education and also learned English at a younger age than did younger adults. Older adults scored higher on the Shipley Institute of Vocabulary Test and on the positive affect scale of the Positive and Negative Affect Schedule (PANAS; Watson et al., 1988), but scored lower on the BAI, the BDI, and the Digit-Symbol Substitution Task (DSST; Wechsler, 1981) than did younger adults. These age differences are typically found in research examining cognitive aging and emotion (e.g., Isaacowitz et al., 2008).

**Table 1:** **Participant characteristics**.

**Measure**	**Younger adults**	**Older adults**
	***M* (*SD*)**	***M* (*SD*)**
Years of education^**^	13.00 (1.74)	17.53 (4.11)
Age learned english^*^	1.13 (1.92)	0.14 (0.83)
Digit-Symbol Substitution Task^a^^**^	82.86 (15.37)	68.78 (14.97)
PANAS-positive affect^b^^**^	27.22 (7.91)	33.83 (6.40)
PANAS-negative affect^b^	15.19 (5.48)	13.61 (4.14)
Shipley vocabulary ^**^	27.50 (3.00)	37.36 (1.93)
Beck Anxiety Inventory^**^	12.67 (7.61)	6.19 (5.67)
Beck Depression Inventory^**^	10.83 (6.67)	5.06 (4.65)
Health rating^c^	7.79 (1.13)	8.25 (1.27)

a Digit-Symbol scores were based on the number of correct solutions within a 2-min time limit;

b PANAS, the Positive and Negative Affect Schedule;

c Health ratings were self-reported based on a 1 (“poor”) to 10 (“excellent”) Likert-type scale;

* *p < 0.01*,

** p < 0.001.

### Stimuli

All stimuli for the delayed-response working memory task in this experiment were programmed with E-prime 1.0 and presented on a 17 inch computer screen. The stimuli consisted of a total of 329 words selected from the Affective Norms of English Words (ANEW) database (Bradley and Lang, 1999). The ANEW database contains normed ratings of arousal (1 for low arousal to 9 for high arousal) and valence (1 for negative valence to 9 for positive valence). Of the 329 words selected, 240 words were used for memoranda (targets or distracters), 20 for new control words, 48 for buffer/filler trials, and 21 for practice trials.

#### Memoranda

A total of 240 words were selected to be targets (*N* = 120) and distracters (*N* = 120) and consisted of 48 positive words (valence *M* = 7.44, range: 6.59–8.39), 48 negative words (valence *M* = 2.86, range: 1.57–3.50), and 144 neutral words (valence *M* = 4.98, range: 4.00–6.00). Half of the words for each valence category were high in arousal (HA; arousal range: 4.51–7.45) and the other half were low in arousal (LA; arousal range: 2.39–4.48), resulting in a total of 6 word lists (one HA and one LA list for each of the three valence categories). All valence categories in the LA list were matched for arousal (*p*s > 0.10); positive and negative HA lists were matched for arousal (*p* = 0.90) and both were higher in arousal than the neutral HA list (*p*s < 0.001). Each of these six lists was then divided into two sets: one set that served as targets and one set that served as distracters. These two sets were matched on word frequency (*M* = 42.21; range 1–294) and word length (i.e., the number of letters) (*M* = 6.26; range: 3–11) (*p*s > 0.21).

Each trial consisted of a set of four memoranda: two target and two distracter words. The combination of words used in each trial varied according to trial type: (1) positive targets paired with neutral distracters (posT/neuD); (2) negative targets paired with neutral distracters (negT/neuD); (3) neutral targets paired with neutral distracters (neuT/neuD); (4) neutral targets paired with positive distracters (neuT/posD); and (5) neutral targets paired with negative distracters (neuT/negD). In each trial, one target/distracter was HA and the other target/distracter was LA. Within each trial, targets and distracters were roughly matched on arousal, frequency, and word length. For example, a neuT/negD trial consisted of one neutral HA target, one neutral LA target, one negative HA distracter, and one negative LA distracter. There were 12 trials for each trial type, resulting in a total of 60 trials. The trials were presented in a pseudorandomized order such that no more than three trials of the same trial type occurred consecutively. The sequencing of memoranda within a trial was also pseudorandomized: half of the trials combined either two targets or two distracters in a row (e.g., distracter-distracter-target-target) and the other half with intermixed targets and distracters (e.g., distracter-target-distracter-target).

#### Probes

After a brief delay following presentation of the memoranda, a probe word was presented. Probes belonged to one of six categories: (1) HA targets; (2) LA targets; (3) HA distracters; (4) LA distracters; (5) HA new control probes; and (6) LA new control probes. Target and distracter probes were from the current trial's memoranda set. New control probes were one of 20 additional words: 4 positive words (valence *M* = 7.41, range: 7.07–7.66), 4 negative words (valence *M* = 2.80, range: 2.73–2.90), and 12 neutral words (valence *M* = 5.08, range: 4.32–5.85), selected from ANEW (Bradley and Lang, 1999); half of the words in each valence category were HA (arousal *M* = 5.70, range: 4.66–6.41) and half were LA (arousal *M* = 3.91, range: 3.18–4.29). The new control probes matched those of the distracters on valence within a trial set (e.g., a neuT/negD trial had a new control probe that was also negative).

The six probe categories occurred equally often for each trial type. The selection of probes (i.e., which target word served as the target probe for a particular trial) was counterbalanced with a Latin Square design, resulting in six counterbalance conditions. In addition, the order within a memoranda set of targets and distracter probes was also counterbalanced such that each appeared equally often at each of the four possible positions (e.g., a HA target cue appeared in the first to fourth position equally).

### Procedure

**Figure 1:**
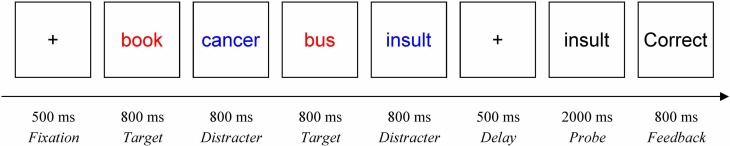
**Working memory task procedure (a neuTnegD trial with a negative distracter probe)**. In this example trial, the participant correctly responded “NO” to the distracter probe and received a feedback screen indicating that the response was correct.

Upon arrival at the laboratory, participants read and signed an informed consent, and then completed the computerized delayed-response working memory task. Participants were seated centrally in front of the computer screen at a reading distance. They were instructed to keep their eyes on the screen at all times during the working memory task. The task instructions indicated which words they should remember (i.e., targets) and which they should ignore (i.e., distracters), as cued by either blue or red font color, counterbalanced across participants. Each trial began with a fixation cross presented for 500 ms, followed by a memoranda set. Each set contained two target and two distracter words, each presented sequentially for 800 ms with an inter-stimulus interval of 200 ms. After a 500 ms delay, a probe word (in black font) was presented for 2000 ms. Participants were instructed to press a key labeled “YES” (the “/” key) if the probe was a target word from the current set. If the probe was a distracter or a new control word, participants were instructed to press the “NO” key (the “Z” key). Participants were instructed to respond as quickly and accurately as possible. Following their responses, an accuracy feedback screen (“Correct,” “Incorrect,” or “No response detected”) was presented for 800 ms (see Figure 1).

The working memory task consisted of a practice block (5 trials; approximately 35 s, with an option to be repeated if needed) and an experimental block (72 trials: 60 experimental, six buffer, and six filler trials; approximately 8.5 min). Buffer trials occurred at the beginning and end of the block to reduce primacy and recency effects and filler trials were randomly intermixed with experimental ones. All buffer and filler trials consisted of target probes, requiring “yes” responses, in order to minimize response bias by balancing the number of “yes” responses with those of “no” responses to make them approximately the same (i.e., 44% “yes” responses). In addition, no more than three “yes” or “no” responses occurred in a row throughout the task.

After the working memory task, participants completed the DSST, a measure of perceptual-motor speed for 2 min. They then completed the PANAS, a self-reported assessment of positive and negative affect. At the end, participants completed a variety of paper-and-pencil tests and questionnaires, including the Shipley Institute of Vocabulary Test, the BAI, the BDI, and a background information questionnaire, to determine their eligibility. Older adults then completed the SBT. Finally, participants were debriefed and paid or granted course credit. The total duration of the experiment was approximately 1 h.

## Results

To examine differential emotional effects of goal-relevant target vs. goal-irrelevant distracting information, response times (RTs) and accuracy in the working memory task were analyzed with mixed analyses of variance (ANOVAs). These analyses were conducted separately for target probe responses (reported as target identification, requiring “yes” responses) and responses to distracter and control probes (reported as interference scores, requiring “no” responses). Only RTs for correct responses were included in the RT analyses. RTs were also trimmed by excluding those that were beyond 2.5 *SD*s away from the mean for each participant in each condition, resulting in an exclusion of 6% of data points. To ensure sufficient correct trials for meaningful RT data in each condition, we replaced three individuals with overall accuracy scores lower than 80% (*M* = 74.33, *SD* = 5.69, range: 68–79).

### Effects of emotional content on target identification

To examine the effects of emotional content (i.e., arousal and valence) on the identification of target probes, a 2 × 2 × 3 mixed ANOVA with Age (younger, older) as a between-subjects variable, Arousal (high, low) and Valence (positive, negative, neutral) as within-subjects variables, was conducted on RTs and accuracy to target probes that require “yes” responses. The data for three younger and two older adults were excluded in the RT analysis because of missing data points due to RT trimming or lack of correct responses in a condition.

#### RT analysis

**Figure 2:**
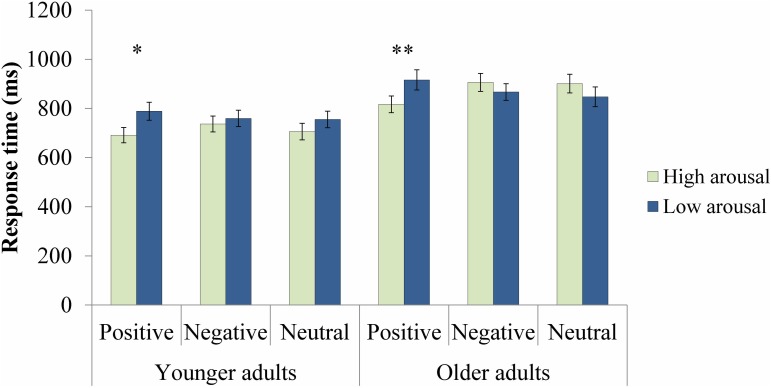
**Response times for correct target identification responses**. Error bars represent standard errors. ^*^p < 0.05; ^**^p < 0.01.

Figure 2 displays the results of the RT analysis. The mixed ANOVA revealed a main effect of Age, *F*_(1, 65)_ = 11.13, *p* = 0.001, η^2^_*p*_ = 0.15. Overall, younger adults (*M* = 739.59, *SD* = 154.08) were faster than older adults (*M* = 875.70, *SD* = 178.61). The main effect of Arousal was significant, *F*_(1, 65)_ = 5.01, *p* = 0.03, η^2^_*p*_ = 0.07. This arousal effect was qualified by an Age by Arousal interaction, *F*_(1, 65)_ = 4.32, *p* = 0.04, η^2^_*p*_ = 0.06. The arousal effect was only significant for younger, *t*_(32)_ = −2.85, *p* = 0.01, but not for older adults (*p* = 0.90). The Arousal by Valence interaction was also significant, *F*_(2, 130)_ = 6.64, *p* = 0.002, η^2^_*p*_ = 0.09, with faster RTs to high arousal than to low arousal for positive, *t*_(66)_ = −4.21, *p* < 0.001, but not for negative (*p* = 0.70) or neutral target probes (*p* = 0.90). This benefit of arousal for positive targets was found for both younger, *t*_(32)_ = −2.54, *p* = 0.02, and older, *t*_(33)_ = −3.59, *p* = 0.001, adults. All other effects were not significant (*F*s < 1.30, *p*s > 0.28).

#### Accuracy analysis

**Figure 3:**
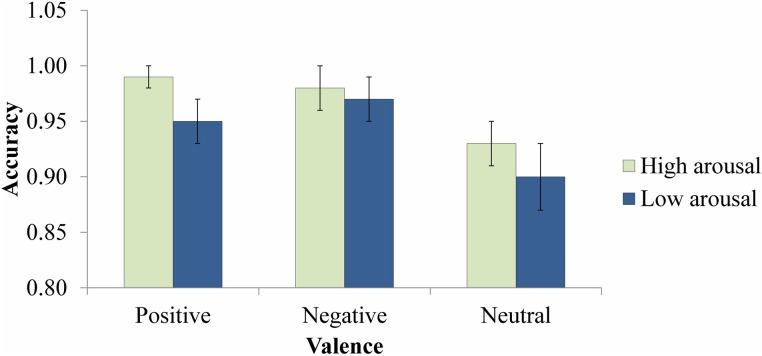
**Accuracy for target identification responses**. Error bars represent standard errors.

The same mixed ANOVA on accuracy revealed a main effect of Valence, *F*_(2, 140)_ = 5.79, *p* = 0.004, η^2^_*p*_ = 0.08, with lower accuracy for neutral (*M* = 0.92, *SD* = 0.16) than for both positive (*M* = 0.97, *SD* = 0.08), *t*_(71)_ = 2.82, *p* = 0.006, and negative words (*M* = 0.97, *SD* = 0.11), *t*_(71)_ = 2.88, *p* = 0.005; the latter two did not differ from each other, *t*_(71) = −0.21_, *p* = 0.84. This suggested a facilitative effect of emotional valence. In addition, the main effect of Arousal was marginally significant, *F*_(1, 70)_ = 3.15, *p* = 0.08, η^2^_*p*_ = 0.04, with higher accuracy for high arousal targets (*M* = 0.97, *SD* = 0.09) than for low arousal targets (*M* = 0.94, *SD* = 0.01), suggesting a trend toward a facilitative effect of emotional arousal (Figure 3). All other effects were not significant (*F*s < 0.31, *p*s > 0.73).

Overall, these findings indicated age-related slowing but preserved accuracy for target identification in working memory, possibly due to age-related shifts toward prioritization of accuracy over speed (Salthouse, 1979). The results also revealed that arousal facilitated the speed in identifying positive targets and valence facilitated target identification accuracy. In summary, emotional content (arousal or valence) facilitated target identification by making responses either faster or more accurate and the effects were largely similar across the two age groups.

### Effects of emotional content on interference of distracters

Following some previous work (Yang and Hasher, 2007), interference was operationally defined as performance differences in responding to distracter vs. matched control probes. This definition is based on the assumption that both distracter and control probes require the same “no” response (i.e., correct rejection of the probe as a target). The primary difference is that distracter probes were presented earlier during encoding. If participants successfully inhibited distracters, we would expect minimal interference at retrieval/probe responding; thus, performance would be very similar to that for control probes (newly presented words). However, if participants were not efficiently inhibiting or deleting the distracter from working memory, these distracter probes may result in false alarms (i.e., incorrectly identifying a distracter as a target probe) or slower rejection responses, thus increasing the performance difference between distracter and the matched control probes.

For the RT analysis, interference was indexed with proportional RT difference scores, calculated by subtracting the RTs to control probes from RTs to the matched distracters (distracters—controls), which was then divided by the RTs to control probes. A similar calculation was applied to the accuracy data, by subtracting the accuracy to distracter probes from that to matched controls, and then divided by the accuracy to controls. Thus, larger scores in both RT and accuracy indicated greater interference. These proportional interference scores controlled for potential age-related differences in response times and accuracy. These interference scores were analyzed with 2 × 2 × 3 mixed ANOVAs with Age (younger, older) as a between-subjects variable, and Arousal (high, low) and Valence (positive, negative, neutral) as within-subjects variables. For the RT analysis only, the data for one younger and four older adults were not included due to missing data points as a result of RT trimming or lack of correct responses in a condition.

#### RT analysis

**Figure 4:**
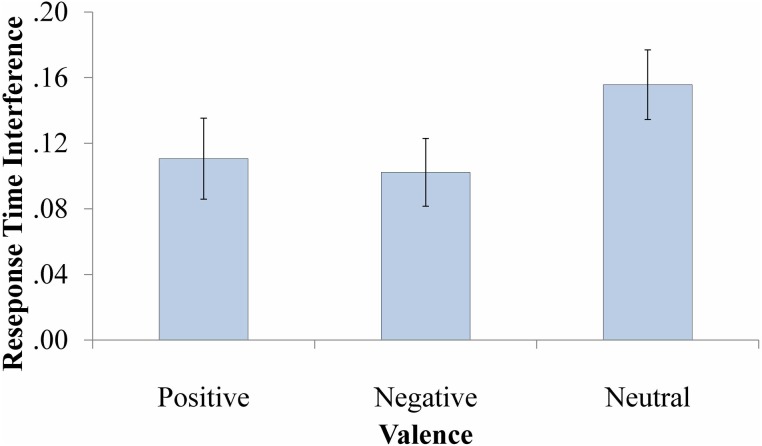
**Response time interference scores**. Error bars represent standard errors.

The mixed ANOVA revealed a marginal main effect of Valence, *F*_(2, 130)_ = 2.62, *p* = 0.08, η^2^_*p*_ = 0.04. Follow-up paired *t*-tests indicated slightly less interference for negative (*M* = 0.10, *SD* = 0.17) vs. neutral (*M* = 0.16, *SD* = 0.17) probes, *t*_(66)_ = −1.79, *p* = 0.08. All other effects were not significant (*F*s < 2.24, *p*s > 0.14). The RT interference scores across valence are displayed in Figure 4.

#### Accuracy analysis

**Figure 5:**
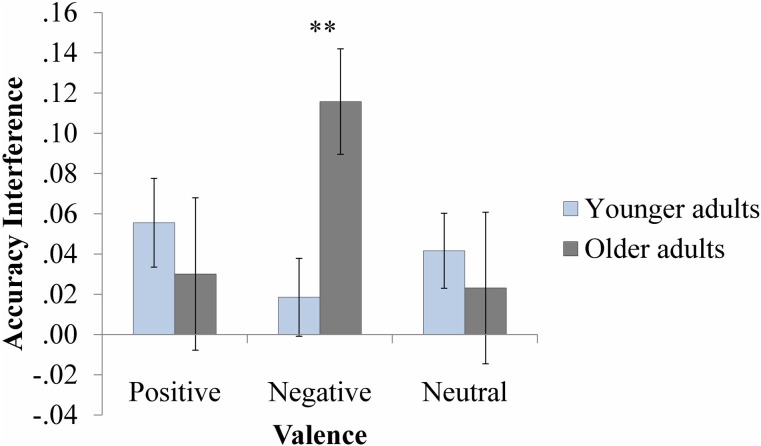
**Accuracy interference scores**. Error bars represent standard errors. ^**^p < 0.01.

Figure 5 displays the results of the accuracy analysis. The ANOVA revealed an Age by Valence interaction, *F*_(2, 140)_ = 3.89, *p* = 0.02, η^2^_*p*_ = 0.05. There was a trend toward a main effect of Valence for older, *F*_(2, 70)_ = 2.49, *p* = 0.09, but not for younger adults, *F*_(1.63, 57.10)_ = 0.83, *p* = 0.42 (Greenhouse-Geisser correction). Guided by our hypotheses, we conducted planned comparisons. The results revealed that older adults experienced greater interference from negative (*M* = 0.12, *SD* = 0.16) than neutral (*M* = 0.02, *SD* = 0.23), *t*_(35)_ = 2.25,*p* = 0.03, and positive probes (*M* = 0.03, *SD* = 0.23), *t*_(35)_ = −1.87, *p* = 0.07; the latter two did not differ, *p* = 0.89. Follow-up independent samples *t*-tests for each valence also indicated age differences for negative probes only: older adults (*M* = 0.12, *SD* = 0.16) experienced greater interference from negative distracters than younger adults (*M* = 0.02, *SD* = 0.12), *t*_(64.49)_ = −2.98, *p* = 0.004. As a matter of fact, older adults experienced significant interference from negative probes, *t*_(35)_ = 4.42, *p* < 0.001, whereas younger adults did not, *t*_(35)_ = 0.96, *p* = 0.35. This age difference was not present for positive or neutral interference scores, *t*s > 0.44, *p*s > 0.56. All other effects were not significant (*F*s < 1.06, *p*s > 0.35).

## Discussion

To our knowledge, this is the first study to examine how the effects of emotional content on working memory vary according to goal relevancy and age. Consistent with our hypotheses, the overall results suggest that working memory is facilitated (i.e., faster and more accurate responses) by the emotional content of goal-relevant target information. In addition, working memory can be disrupted (i.e., larger interference) by the emotional content of goal-irrelevant distraction. However, this disruptive effect appears to be limited to negatively-valenced stimuli and occurs only for older adults. These results add novel contributions to our understanding of facilitative and disruptive emotional effects on working memory by manipulating and distinguishing between goal-relevant and goal-irrelevant information.

### Emotional target information facilitates working memory

The dual-competition model (DCM; Pessoa, 2008, 2009) posits that emotional information receives prioritized processing, which can be beneficial when the information is relevant to current goals. Our study found support for this prediction as responses were faster or more accurate to emotional/high arousing relative to neutral/low arousing goal-relevant target words. These results are consistent with studies that have found facilitative effects of emotion on working memory (Mammarella et al., 2013a). However, the facilitative effect of arousal only occurred for response speed, and was significant only for positive, but not for negative, target words. This appears to conflict with predictions from the DCM of enhanced processing for high arousal negative information (e.g., threat-relevant stimuli). We advise caution when interpreting this result, as performance was close to ceiling for negative targets, which may have limited our ability to find differences in performance between low and high arousal negative words. However, the findings of faster responses to positive high arousal targets are consistent with another study in which high arousal positive targets were detected faster amidst an array of neutral distracters (Leclerc and Kensinger, 2008). Taken together, it appears that high arousal positive goal-relevant stimuli can receive prioritized processing in competition against neutral distracters in the current paradigm.

In addition, the results indicated higher accuracy for emotional vs. neutral target word identification for both younger and older adults. There was also a pattern toward more accurate responses for high vs. low arousal words. These results are consistent with our hypotheses and other findings of attenuation of age differences when emotional materials are used (e.g., Mikels et al., 2005; Borg et al., 2011; Mammarella et al., 2013a). However, we found less evidence of a specific advantage for positive goal-relevant information in older adults, which we hypothesized based on literature pertaining to the positivity effect. Instead, our overall results indicated that emotional content in general, regardless of valence, appears to help younger and older adults to a similar extent. Together, these results may suggest that older adults are still capable of allocating resources to prioritize processing of emotional items, and thus show no impairment on working memory accuracy for target information.

### Negative distracter information disrupts working memory in older adults

In contrast to the facilitative effects of emotion on working memory, there was some evidence supporting our hypothesis that emotional goal-irrelevant information can disrupt performance. Negative distracter stimuli caused greater interference in working memory, resulting in lower accuracy performance, but this was found for older adults only; no such effect occurred for younger adults. This finding converges, to some degree, with a large body of literature that suggests a shift toward a positivity bias in older adults vs. a negativity bias in younger adults (Reed and Carstensen, 2012). Although we hypothesized that older adults' enhanced attention to positive distracters would be detrimental to their working memory performance, our results suggested poorer performance due to negative distracters. Older adults were also less successful at resolving interference from negative distracters compared to younger adults. Poorer interference resolution involving negative distracters in older adults relative to younger adults is in line with research suggesting that younger and older adults differ in how negative information is processed, with greater attention toward negative information in younger adults and avoidance of negative information in older adults (Mather and Carstensen, 2003; Isaacowitz et al., 2006). A speculative interpretation of these findings may be derived based on the dual-competition model (DCM; Pessoa, 2008, 2009). DCM posits that task-irrelevant emotional stimuli can impair executive control by consuming resources needed for conflict resolution. If older adults have an “anti-negativity” bias, defined as avoidance of negative, relative to younger adults (e.g., Isaacowitz et al., 2006; Knight et al., 2007) or have particular difficulties processing cognitively-demanding negative information (Labouvie-Vief et al., 2010), it is possible that they did not devote sufficient resources to successfully encode negative words as distracters. This would result in a weaker representation of the negative word as a distracter item. When they encountered these words as probes during retrieval, resources may have been reallocated to prioritize the processing of these weak representations of negative information, at the expense of interference resolution. Thus, older adults' poorer performance with negative interference may be due to a “double-edged sword” of negative goal-irrelevant information, caused by both anti-negativity biases during encoding and competition between emotional information and executive control for limited resources at retrieval. In contrast, younger adults may have more resources to spare and appear to be more successful in processing negative content while simultaneously engaging in interference resolution. This is demonstrated in our study and others (Levens and Phelps, 2008) as an absence or reduction of interference arising from negative probes for younger adults.

Alternatively, disrupted performance from negative interference in older adults could also be explained as heightened attention to negative information in older adults. A negativity bias in older adults has been observed when cognitive resources are limited, often manipulated through divided or dual attention paradigms (Mather and Knight, 2005; Knight et al., 2007). Thus, it is possible that the working memory paradigm used in the current study sufficiently reduced or divided cognitive resources in older adults by requiring interference resolution, which resulted in greater attention toward negative information. This enhanced processing of negative information may have distracted older adults to a greater extent relative to positive and neutral information, resulting in increased false alarms to negative distracter probes.

### Future outlook

Taken together, the results from this study provide evidence suggesting that the differential effects of emotion on working memory may vary by arousal, valence, age, and goal relevancy. However, there were several limitations of the current study. First, the irrelevant information in the working memory task (i.e., distracter words) was arbitrarily and externally assigned by experimenters. Thus, this paradigm does not inform us about the interference resolution of internally generated distractions, such as in proactive interference paradigms or when there is a mismatch between task goals and emotion regulation goals. Future research could address this question by modifying the paradigm to include internally generated distracter items to measure the impact of these items on working memory. Furthermore, it could also manipulate the match/mismatch of task vs. emotional goals by implementing task instructions that explicitly direct participants to engage in emotional processing, which contrasts with the more perceptually-based processing used in the current paradigm (i.e., cuing of target vs. distracter via different colored fonts).

The second limitation is our sample. The older sample has a larger age range (i.e., 22 years) than the younger sample (i.e., 11 years). This wider range in older adults may have introduced larger performance variability (e.g., Hultsch et al., 2002). We assume that any variability in the older adult group may also be reflective of aging more generally, as studies typically find that both inter- and intra-individual variability increases with age (e.g., Nelson and Dannefer, 1992; Shammi et al., 1998). As this study was a preliminary examination of emotion on working memory and aging, to better encompass any changes that may occur in later life, we followed a common approach in cognitive aging literature and did not set an upper age limit for the older group. Additionally, the low percentage of male participants (13% of the total sample) was another limitation of this sample. In consideration of research on gender differences in emotion regulation and processing (e.g., Gur and Gur, 2002; McRae et al., 2008), our findings may not be representative of emotion processing in males. As such, future studies should aim for more balanced numbers of female and male participants.

Finally, although we expected relatively high accuracy on this task based on results from other studies (e.g., Gazzaley et al., 2005), we also expected to find some evidence of age-related working memory declines. Aside from slower target identification, which could be attributed to age-related prioritization of accuracy over speed, there were no main effects of age. The lack of age differences suggests that this paradigm may not exert high demands on working memory. This may have limited our ability to find facilitative or disruptive effects of emotion. However, the lack of age differences may also suggest that the emotional nature of the task may have helped attenuate overall age-related working memory declines, as has been found in other studies (e.g., Mikels et al., 2005). To examine this, future studies could use a working memory paradigm that is more taxing and thus more sensitive at detecting age differences. Future research could also build on the results of this study by examining the neural mechanisms that contribute to the facilitative and disruptive effects of emotion in the aging brain. Such work could be informed by research on younger adults (Dolcos et al., 2011) that have identified multiple neural connections between areas implicated in emotion processing (e.g., ventral affective system) and cognitive control (e.g., dorsal executive system).

Overall, this study provided novel evidence in support of recent frameworks that specify the competitive advantage of emotional over non-emotional information (particularly for older adults; Carstensen et al., 1999) and the role of goal relevancy (Pessoa, 2008, 2009). It contributed to the sparse, but growing, literature on the important interactions between emotion and cognitive control in older adults (e.g., Pessoa, 2008; Dolcos et al., 2011). Such work helps identify situations in which older adults' preserved emotional processing could be a helpful “friend” vs. hindering “foe” to their declines in cognitive control.

### Conflict of interest statement

The authors declare that the research was conducted in the absence of any commercial or financial relationships that could be construed as a potential conflict of interest.

## References

[B1] Beck A. T. Epstein N. Brown G. Steer R. A. (1988). An inventory for measuring clinical anxiety: psychometric properties. J. Consult. Clin. Psychol. 56, 893–897 10.1037/0022-006X.56.6.893 3204199

[B2] Beck A. T. Steer R. A. Brown G. K. (1996) Manual for the Beck Depression Inventory-II. San Antonio, TX: Psychological Corporation

[B3] Borg C. Leroy N. Favre E. Laurent B. Thomas-Antérion C. (2011). How emotional pictures influence visuospatial binding in short-term memory in ageing and Alzheimer's disease? Brain Cogn. 76, 20–25 10.1016/j.bandc.2011.03.008 21481999

[B4] Bradley M. M. Lang P. J. (1999). Affective norms for English words (ANEW): stimuli, instruction manual and affective ratings, in Technical Report C-1 (Gainesville, FL: The Center for Research in Psychophysiology, University of Florida).

[B5] Carstensen L. L. Isaacowitz D. M. Charles S. T. (1999). Taking time seriously: a theory of socioemotional selectivity. Am. Psychol. 54, 165–181 10.1037/0003-066X.54.3.165 10199217

[B6] Döhnel K. Sommer M. Ibach B. Rothmayr C. Meinhardt J. Hajak G. (2008). Neural correlates of emotional working memory in patients with mild cognitive impairment. Neuropsychologia 46, 37–48 10.1016/j.neuropsychologia.2007.08.012 17915264

[B7] Dolcos F. Iordan A. Dolcos S. (2011). Neural correlates of emotion–cognition interactions: a review of evidence from brain imaging investigations. J. Cogn. Psychol. 23, 669–694 10.1080/20445911.2011.594433 22059115 PMC3206704

[B8] Dolcos F. McCarthy G. (2006). Brain systems mediating cognitive interference by emotional distraction. J. Neurosci. 26, 2072–2079 10.1523/JNEUROSCI.5042-05.2006 16481440 PMC6674921

[B9] Gazzaley A. Cooney J. W. Rissman J. D'Esposito M. (2005). Top-down suppression deficit underlies working memory impairment in normal aging. Nat. Neurosci. 8, 1298–1300 10.1038/nn1543 16158065

[B10] Gur R. E. Gur R. C. (2002). Gender differences in aging: cognition, emotions, and neuroimaging studies. Dialogues Clin. Neurosci. 4, 197–210 22033483 10.31887/DCNS.2002.4.2/rgur PMC3181676

[B11] Hasher L. Zacks R. T. (1988). Working memory, comprehension, and aging: a review and a new view, in The Psychology of Learning and Motivation: Advances in Research and Theory, Vol. 22, ed Bower G. H. (New York, NY: Academic Press), 193–225

[B12] Hultsch D. F. MacDonald S. W. S. Dixon R. A. (2002). Variability in reaction time performance of younger and older adults. J. Gerontol. B Psychol. Sci. Soc. Sci. 57, 101–115 10.1093/geronb/57.2.P101 11867658

[B13] Isaacowitz D. M. Toner K. Goren D. Wilson H. R. (2008). Looking while unhappy: mood-congruent gaze in young adults, positive gaze in older adults. Psychol. Sci. 19, 848–853 10.1111/j.1467-9280.2008.02167.x 18947348 PMC2760922

[B14] Isaacowitz D. M. Wadlinger H. A. Goren D. Wilson H. R. (2006). Is there an age-related positivity effect in visual attention? A comparison of two methodologies. Emotion 6, 511–516 10.1037/1528-3542.6.3.511 16938091

[B15] Katzman R. Brown T. Fuld P. Peck A. Schechter R. Schimmel H. (1983). Validation of a short orientation-memory-concentration test of cognitive impairment. Am. J. Psychiatry 140, 734–739 6846631 10.1176/ajp.140.6.734

[B16] Kensinger E. A. Corkin S. (2004). Two routes to emotional memory: distinct neural processes for valence and arousal. Proc. Natl. Acad. Sci. U.S.A. 101, 3310–3315 10.1073/pnas.0306408101 14981255 PMC365786

[B17] Knight M. Seymour T. L. Gaunt J. T. Baker C. Nesmith K. Mather M. (2007). Aging and goal-directed emotional attention: distraction reverses emotional biases. Emotion 7, 705–714 10.1037/1528-3542.7.4.705 18039037

[B18] Labouvie-Vief G. (2003). Dynamic integration: affect, cognition, and the self in adulthood. Curr. Dir. Psychol. Sci. 12, 201–206 10.1046/j.0963-7214.2003.01262.x

[B19] Labouvie-Vief G. Grühn D. Studer J. (2010). Dynamic integration of emotion and cognition: equilibrium regulation in development and aging, in The Handbook of Lifespan Development, eds Lerner R. M. Lamb M. E. Freund A. M. (Hoboken: John Wiley and Sons, Inc.), 79–115

[B20] Leclerc C. M. Kensinger E. A. (2008). Effects of age on detection of emotional information. Psychol. Aging 23, 209–215 10.1037/0882-7974.23.1.209 18361668

[B21] Levens S. M. Phelps E. A. (2008). Emotion processing effects on interference resolution in working memory. Emotion 8, 267–280 10.1037/1528-3542.8.2.267 18410200

[B22] Mammarella N. Borella E. Carretti B. Leonardi G. Fairfield B. (2013a). Examining an emotion enhancement effect in working memory: evidence from age-related differences. Neuropsychol. Rehabil. 23, 416–428 10.1080/09602011.2013.775065 23452136

[B23] Mammarella N. Fairfield B. Di Domenico A. Borella E. Carretti B. (2013b). Is working memory affective in dementia of alzheimer's type? Neurosci. Discov. 1, 1–4 10.7243/2052-6946-1-4

[B24] Mather M. Carstensen L. L. (2003). Aging and attentional biases for emotional faces. Psychol. Sci. 14, 409–415 10.1111/1467-9280.01455 12930469

[B25] Mather M. Carstensen L. L. (2005). Aging and motivated cognition: the positivity effect in attention and memory. Trends Cogn. Sci. 9, 496–502 10.1016/j.tics.2005.08.005 16154382

[B26] Mather M. Knight M. (2005). Goal-directed memory: the role of cognitive control in older adults' emotional memory. Psychol. Aging 20, 554–570 10.1037/0882-7974.20.4.554 16420131

[B27] McRae K. Ochsner K. N. Mauss I. B. Gabrieli J. J. D. Gross J. J. (2008). Gender differences in emotion regulation: an fMRI Study of cognitive reappraisal. Gr. Process. Intergr. Relat. 11, 143–162 10.1177/1368430207088035 29743808 PMC5937254

[B28] Mikels J. A. Larkin G. R. Reuter-Lorenz P. A. Cartensen L. L. (2005). Divergent trajectories in the aging mind: changes in working memory for affective versus visual information with age. Psychol. Aging 20, 542–553 10.1037/0882-7974.20.4.542 16420130 PMC2746384

[B29] Murphy N. A. Isaacowitz D. M. (2008). Preferences for emotional information in older and younger adults: a meta-analysis of memory and attention tasks. Psychol. Aging 23, 263–286 10.1037/0882-7974.23.2.263 18573002

[B30] Nelson E. A. Dannefer D. (1992). Aged heterogeneity: fact or fiction? the fate of diversity in gerontological research. Gerontologist 32, 17–23 10.1093/geront/32.1.17 1740251

[B31] Pessoa L. (2008). On the relationship between emotion and cognition. Nat. Rev. Neurosci. 9, 148–158 10.1038/nrn2317 18209732

[B32] Pessoa L. (2009). How do emotion and motivation direct executive control? Trends Cogn. Sci. 13, 160–166 10.1016/j.tics.2009.01.006 19285913 PMC2773442

[B33] Reed A. E. Carstensen L. L. (2012). The theory behind the age-related positivity effect. Front. Psychol. 3:339 10.3389/fpsyg.2012.00339 23060825 PMC3459016

[B34] Rypma B. D'Esposito M. (2000). Isolating the neural mechanisms of age-related changes in human working memory. Nat. Neurosci. 3, 509–515 10.1038/74889 10769393

[B35] Salthouse T. A. (1979). Adult age and the speed-accuracy trade-off. Ergonomics 22, 811–821 10.1080/00140137908924659 488072

[B36] Scheibe S. Carstensen L. L. (2010). Emotional aging: recent findings and future trends. J. Gerontol. Psychol. Sci. 65B, 135–144 10.1093/geronb/gbp132 20054013 PMC2821944

[B37] Shammi P. Bosman E. Stuss D. T. (1998). Aging and variability in performance. Aging Neuropsychol. Cogn. 5, 1–13 10.1076/anec.5.1.1.23

[B38] Shipley W. C. (1940). A self-administering scale for measuring intellectual impairment and deterioration. J. Psychol. Interdiscip. Appl. 9, 371–337 10.1080/00223980.1940.9917704

[B39] Watson D. Clark L. A. Tellegen A. (1988). Development and validation of brief measures of positive and negative affect: the PANAS scales. J. Pers. Soc. Psychol. 54, 1063–1070 10.1037/0022-3514.54.6.1063 3397865

[B40] Wechsler D. (1981). Manual for the Wechsler Adult Intelligence Scale-Revised. New York, NY: Psychological Corporation

[B41] Yang L. Hasher L. (2007). The enhanced effects of pictorial distraction in older adults. J. Gerontol. B. Psychol. Sci. Soc. Sci. 62, 230–233 10.1093/geronb/62.4.P230 17673533

